# Dry Needling in Treatment of Temporomandibular Joint Disorders: A Systematic Review

**DOI:** 10.1002/cre2.70214

**Published:** 2025-09-08

**Authors:** Mina Khayamzadeh, Farnoosh Razmara, Afagh Tavassoli

**Affiliations:** ^1^ Department of Oral and Maxillofacial Medicine Tehran University of Medical Sciences, International Campus Tehran Iran; ^2^ Craniomaxillofacial Research Center Tehran University of Medical Sciences Tehran Iran; ^3^ Department of Oral and Maxillofacial Surgery, School of Dentistry Tehran University of Medical Sciences Tehran Iran; ^4^ Universal Scientific Education and Research Network (USERN) Tehran Iran

**Keywords:** dry needling, pain, temporomandibular joint disorders

## Abstract

**Objectives:**

Among the minimally invasive techniques for treating temporomandibular joint disorders (TMDs) is dry needling, which can be used as a potential treatment method. This study aims to review current knowledge to understand the impact of dry needling on treating TMDs.

**Methods:**

This systematic review was carried out in alignment with the guidelines outlined in the Preferred Reporting Items for Systematic Reviews and Meta‐Analyses (PRISMA). A comprehensive literature search was performed using PubMed, Scopus, and Google Scholar. The search was done on the studies published between 2000 and 2024. Cochrane Collaboration's Risk of Bias tool for randomized controlled trials (RCTs) was applied to evaluate the risk of bias.

**Results:**

A total of 673 studies were identified. Among these, 245 articles were assessed for eligibility; ultimately, 10 studies were made up of the final review. These studies evaluated several outcome measures, the most common of which were: the visual analog scale (VAS), verbal rating scale (VRS), electromyography (EMG), extent of mouth opening, pain symptomatology, myofascial trigger point pain, sonographic measurements, bilateral muscle palpation with a pressure algometer, Tinnitus Handicap Inventory (THI), and mandibular mobility. Most RCTs had a low risk of bias.

**Conclusion:**

The findings consistently underscore the role of dry needling (DN) and other adjunctive therapies in improving clinical outcomes, particularly pain reduction and functional improvement.

## 1 Introduction

Temporomandibular joint disorders (TMDs) include different musculoskeletal and neuromuscular conditions that can influence the temporomandibular joint (TMJ), mouth muscles, and other organs associated with it. These disorders are highly prevalent, with studies showing that ~5%–12% of the population seek treatment due to the severity of their symptoms (Manfredini et al. 2010). Pain and limitation in jaw movement, joint noises, and popping are among the most common symptoms of this disorder, which can affect people's regular functions and quality of life (Okeson 2019).

The etiological cause of this disorder is multifactorial, including psychosocial, neuromuscular, and biomechanical factors (Leeuw and Klasser 2018). Treatment protocols for this disorder focus on pharmacological interventions, physical therapy, behavioral therapies, and occlusal devices. Although these approaches and strategies have not satisfied all patients, they have decreased the need for alternative or adjunctive therapies (Conti et al. 2015).

One of the minimally invasive techniques for treating this disorder is dry needling, which can be used as a potential treatment method. In this technique, fine needles are inserted into the body at myofascial trigger points to reduce pain, maintain function, and improve organ movement (Dommerholt et al. 2006). In addition to this disorder, this method is widely used in back pain, myofascial pain syndrome, and whiplash. Moreover, acupuncture, a form of complementary and alternative medicine, has been implemented in China for more than 3000 years, where it has been traditionally applied to control chronic pain (Valera‐Calero et al. 2022). There is scientific proof that dry needling can reduce pain and lead to functional improvement in TMD cases (Kietrys et al. 2014). Although its use is still developing, the exact mechanisms that can explain the therapeutic effects are still being studied. These mechanisms include local muscle relaxation, improved blood flow to the affected areas, and reduction and modulation of pain pathways (Barbero et al. 2019).

This study will be focused on the role of dry needling in the treatment of TMD. To this end, we evaluated the clinical effectiveness of the mechanisms of action and their integration into treatment protocols from the perspective of different articles. Therefore, this study is a survey of the existing knowledge on the impact of dry needling in the treatment of TMD.

## 2 Materials and Methods

### 2.1 Study Design

**Figure 1:**
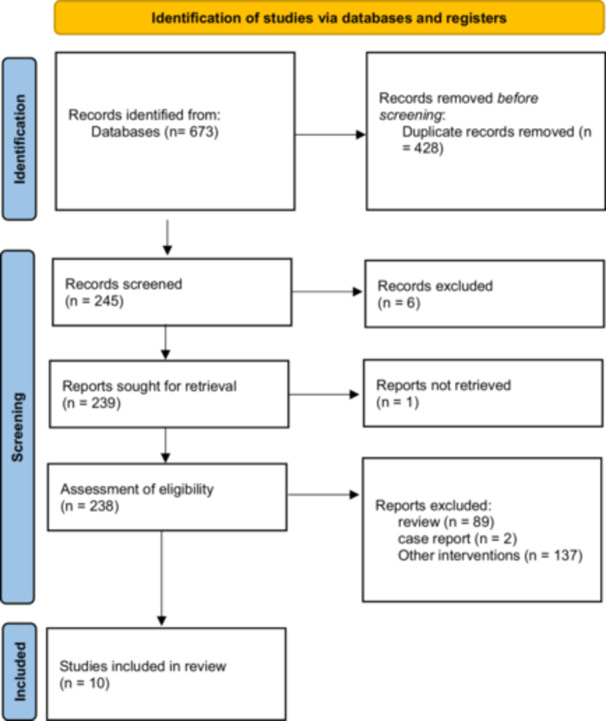
Flowchart for literature related to this systematic review.

This systematic review was carried out with the consideration of the guidelines outlined in the Preferred Reporting Items for Systematic Reviews and Meta‐Analyses (PRISMA) (Page et al. 2021) (Figure 1).

### 2.2 Eligibility Criteria

Included criteria for the study were considered as follows:

- Evaluated the utilization of dry needling as a treatment for TMD.
- Involved adults (18 years and older) with diagnosed TMD.
- Employed randomized controlled trials (RCTs) and clinical trials.
- Reported outcomes related to pain reduction, jaw mobility, and quality of life.

Moreover, exclusion criteria were as follows: case reports, reviews, or studies with insufficient data or outcomes.

### 2.3 Information Sources

A comprehensive literature search was executed using PubMed, Scopus, and Google Scholar. The search was limited to studies published between 2000 and 2024, and the literature review had been completed by the first of February 2025. Also, to ensure a comprehensive approach, the literature review was not restricted by language, and all relevant publications, regardless of their origin, were considered for inclusion.

### 2.4 Search Strategy

The search strategy was based on these keywords: “dry needling,” “temporomandibular joint disorders,” “TMD,” “myofascial pain,” and “musculoskeletal pain.”

### 2.5 Study Selection

Two independent reviewers screened all studies, focusing on the titles and abstracts to assess eligibility. All studies were reviewed for inclusion by reading the main text, and disagreements were resolved through a third reviewer.

### 2.6 Data Extraction

Two reviewers extracted the data independently by implementing a data extraction form. The following data were collected:

- Study characteristics (author, country).
- Patient demographics (age, sample size).
- Intervention details (protocol of dry needling treatment, frequency, duration).
- Outcome measures (pain scores, range of motion).

### 2.7 Risk of Bias Assessment

The risk of bias was evaluated by the Cochrane Collaboration's Risk of Bias tool for RCTs (Sterne et al. 2019) (Table 1). The risk of bias table assesses the quality of the methodology of the included studies. Following is a description of the risk‐of‐bias table based on the Cochrane Risk of Bias tool for RCTs. The items assessed for each of the studies are as follows: sequence generation, allocation concealment, blinding of participants and personnel, blinding of outcome assessment, incomplete outcome data, selective reporting, and other bias.

**Table 1:** Risk of bias for the included studies.

References	Sequence generation	Allocation concealment	Blinding of participants and personnel	Blinding of outcome assessment	Incomplete outcome data	Selective reporting	Other bias	Risk of bias
Davydenko (2024)	L^a^	U^b^	L	L	L	L	L	L
Sekito et al. (2022)	L	L	L	L	L	L	L	L
Reis et al. (2017)	L	U	U	U	L	L	L	L
Oliveira et al. (2018)	L	U	U	U	L	L	L	L
Fouda (2014)	L	L	L	L	L	L	L	L
Dunning et al. (2024)	L	L	L	L	L	L	L	L
Botticchio et al. (2021)	L	L	L	U	L	L	L	L
Dib‐Zakkour et al. (2022)	L	L	L	L	L	L	L	L
Sirikaku et al. (2023)	L	L	L	L	U	L	L	L
Ferreira et al. (2024)	L	L	L	L	U	L	L	L

^a^ Low.

^b^ Unclear.

Each of these items is rated as “Low,” “High,” or “Unclear” risk depending on the quality of reporting and evidence. Finally, a qualitative synthesis of the included studies was accomplished.

## 3 Results

### 3.1 Study Selection

A total of 673 studies were identified through the systematic search of the databases PubMed, Scopus, and Google Scholar. Duplicates were removed, and 428 studies were evaluated for eligibility. Six studies were excluded based on title and abstract review due to irrelevance or failure to meet the inclusion criteria. A total of 245 articles were assessed for eligibility, and ultimately, 10 studies were included in the final review. The study selection procedure is summarized in the PRISMA flowchart (Figure 1).

### 3.2 Study Characteristics

The characteristics of the included studies are summarized in Table 2. The studies included ten RCTs. The sample sizes varied from 10 to 120 participants. The mean age of contributors across the studies ranged from 18 to 70 years. The interventions were variable, with dry needling being applied to different muscle groups, including the masseter, temporalis, and other masticatory muscles.

**Table 2:** Characteristics of the included studies in the review.

References	Age	Sample size	Study population	Study groups	Outcomes	Interpretation
Davydenko (2024)	Adults > 18	44	Ukraine	Experimental: deep puncture of trigger points; Control: sham intervention	Myofascial pain, VAS^a^ scores, trigger point count	VAS scores dropped by 63.51% in the experimental group and by 25.71% in controls. Trigger points decreased by 57.09% and 1.31%, respectively, demonstrating the short‐term effectiveness of DN for pain and trigger point reduction.
Sekito et al. (2022)	45 ± 13 years	28	Brazil	Group 1: fascial manipulation; Group 2: conventional TMD^b^ treatment (occlusal appliance, anesthetics, DN^c^)	VRS^d^, RDC/TMD^e^, EMG^f^, pain/pressure evaluation	Both groups improved on VRS (*p* < 0.0001) and pain‐free opening (*p* < 0.001), but Group 1 recovered faster, making facial manipulation a cost‐effective option for TMD management.
Reis et al. (2017)	39 years	10	Brazil	Weekly DN sessions targeting masseteric trigger points	VAS scores, maximum mouth‐opening	VAS scores improved significantly (8.3–2.3, *p* < 0.05). Mouth opening increased, but without statistical significance (*p* > 0.05), supporting DN as a viable treatment for myofascial pain.
Oliveira et al. (2018)	18–70 years	10	Brazil	G1: Low‐level laser therapy; G2: Dry needling	Pain symptoms, mouth opening	Both groups improved (*p* < 0.05). DN showed slightly faster progress in mouth opening, though not statistically significant. Both therapies were effective for myofascial pain relief.
Fouda (2014)	30 years	72	Egypt	Four groups: low‐level laser, DN, anesthetic injections, PEMF^g^	Trigger point pain	PEMF emerged as the most effective for pain relief, suggesting its use as an adjunct to other therapies for trigger point management.
Dunning et al. (2024)	40.2 ± 12.4 years	120	USA	Group 1: DN + spinal manipulation; Group 2: Splint + NSAIDs^h^ + mobilization	VAS scores, mouth opening	DN with spinal manipulation significantly reduced pain and increased pain‐free mouth opening at 3 months compared to conventional therapy (*p* < 0.001).
Botticchio et al. (2021)	22 ± 1.9 years	17	Spain	Pre‐ and post‐DN sonographic evaluations	Disc/muscle thickness, mouth opening	DN reduced disc/muscle thickness and improved mouth opening, indicating positive morphological and functional effects.
Dib‐Zakkour et al. (2022)	18–40 years	36	Spain	Group E: bilateral DN; Group C: sham technique	VAS scores, muscle palpation, jaw movement	DN reduced facial pain and improved jaw symmetry, muscle activity, and mouth opening. Positive effects on muscle function and trajectory were observed.
Sirikaku et al. (2023)	Not specified	25	Brazil	Group 1: DN; Group 2: sham DN	VAS, Tinnitus Handicap Inventory (THI)	DN reduced tinnitus intensity and discomfort more effectively than sham (*p* = 0.041). Benefits were maintained over 90 days, supporting DN's multidimensional efficacy.
Ferreira et al. (2024)	Not specified	60	Brazil	Group 1: DN and manual therapy; Group 2: Cognitive‐behavioral therapy	VAS scores, mandibular mobility	DN and manual therapy significantly improved pain and mandibular mobility (*p* < 0.001). Large effect sizes confirmed strong therapeutic value.

^a^ Visual analog scale.

^b^ Temporomandibular joint disorders.

^c^ Dry needling.

^d^ Verbal rating scale.

^e^ Research diagnostic criteria for temporomandibular joint disorders.

^f^ Electromyography.

^g^ Pulsed electromagnetic fields.

^h^ Nonsteroidal anti‐inflammatory drugs.

### 3.3 Outcome Measures

The included studies evaluated several outcome measures, the most common of which were: the visual analog scale (VAS) (Davydenko 2024; Reis et al. 2017; Dunning et al. 2024; Dib‐Zakkour et al. 2022; Sirikaku et al. 2023; Ferreira et al. 2024), verbal rating scale (VRS), electromyography (EMG) (Sekito et al. 2022), the extent of mouth opening (Reis et al. 2017; Oliveira et al. 2018; Botticchio et al. 2021), pain symptomatology (Oliveira et al. 2018), myofascial trigger point's pain (Fouda 2014), sonographic measurements (Botticchio et al. 2021), bilateral muscle palpation with a pressure algometer (Dib‐Zakkour et al. 2022), Tinnitus Handicap Inventory (THI) (Sirikaku et al. 2023), and mandibular mobility (Ferreira et al. 2024).

### 3.4 Risk of Bias

The risk of bias in the included studies is summarized in Table 1. Most RCTs had a low risk of bias in sequence generation and allocation concealment. However, they showed a moderate to high risk of bias in the blinding of contributors and outcome assessment as a result of the essence of the intervention. Finally, all included studies have a low risk of bias.

### 3.5 Heterogeneity

The heterogeneity across the included studies was relatively high, primarily due to differences in intervention protocols and outcome measures. Therefore, a meta‐analysis was not performed for these studies.

### 3.6 Overall Clinical Procedure

Muscles were examined manually or with a ballpoint pen to find any painful taut bands or trigger points (TP), and then the skin was disinfected with 70% alcohol (Reis et al. 2017; Dib‐Zakkour et al. 2022; Sirikaku et al. 2023; Botticchio et al. 2021; Fouda 2014). Sterilized disposable stainless steel acupuncture needles with a diameter of 0.18–0.3 mm and a length of 13–60 mm were used for DN (Reis et al. 2017; Dunning et al. 2024; Dib‐Zakkour et al. 2022; Sirikaku et al. 2023; Ferreira et al. 2024; Oliveira et al. 2018; Botticchio et al. 2021). The overlying skin was fixed between the index finger and middle finger or the index finger and thumb (Sirikaku et al. 2023; Fouda 2014). In the next step, the needle was inserted perpendicular to the muscles to reach TPs. The depth of insertion varied between 10 and 35 mm, depending on the different anatomical targets. After placing the needle, rotary movements or bidirectional manipulation were implemented to create local responses such as aching, tingling, deep pressure feeling, warmth, or heaviness (Reis et al. 2017; Dunning et al. 2024; Sirikaku et al. 2023; Oliveira et al. 2018; Botticchio et al. 2021). The time that the needle was left in each TP was reported between 10 s and 30 min in different studies (Reis et al. 2017; Dunning et al. 2024; Ferreira et al. 2024; Oliveira et al. 2018; Botticchio et al. 2021; Fouda 2014). Most studies applied this technique weekly for 3–6 weeks (Reis et al. 2017; Dunning et al. 2024; Sirikaku et al. 2023; Sekito et al. 2022; Fouda 2014).

## 4 Discussion

This review evaluated the impact of various therapeutic modalities for managing myofascial pain and TMD. The findings consistently underscore the role of DN and other adjunctive therapies in improving clinical outcomes, particularly pain reduction and functional improvement.

### 4.1 Effectiveness of Dry Needling

Dry needling emerged as a highly effective modality for reducing pain intensity and enhancing functional outcomes in TMD cases. Davydenko (2024) indicated significant reductions in VAS scores (63.51%) and trigger point quantity (57.09%) in the experimental group compared to the control, highlighting the short‐term efficacy of DN. Similarly, Reis et al. (2017) reported a statistically significant decline in VAS scores (from 8.3 to 2.3, *p* < 0.05) following DN sessions in the masseteric region, suggesting DN as a viable alternative for managing myofascial pain.

Further corroborating this, Dunning et al. (2024) found that DN combined with spinal manipulation yielded superior pain relief and mouth opening compared to interocclusal splint therapy, pharmacological management, and nonthrust joint mobilization technique at the 3‐month follow‐up (*p* < 0.001). These findings collectively demonstrate that DN reduces pain and promotes functional recovery in patients with TMD.

Sekito's study evaluated the effectiveness of DN combined with anesthesia injection and occlusal appliance therapy compared to fascial manipulation (FM), and it demonstrated that both treatment plans can result in a significant pain reduction, while FM promotes faster function recovery (Sekito et al. 2022).

The therapeutic effects of DN extend to addressing associated symptoms of TMD, such as tinnitus. Sirikaku et al.'s (2023) study revealed that DN, when combined with counseling, significantly reduced tinnitus intensity and discomfort, alongside improvements in pain and mandibular mobility. This multidimensional efficacy underscores the DN's versatility in managing TMD and its related symptoms.

Botticchio et al. (2021) highlighted the morphological changes induced by DN, such as reduced muscle and articular disc thickness, alongside improvements in maximum mouth opening. These findings suggest that DN may positively influence both structural and functional parameters, while further investigations are recommended to evaluate the possibility of correlation between morphological changes and functional outcomes. Additionally, Dib‐Zakkour et al. (2022) emphasized that DN facilitates enhanced jaw symmetry and trajectory during movement, further supporting its role in improving biomechanical alignment and functional outcomes.

The safety profile and noninvasive nature of DN add to its appeal as a treatment modality. Multiple studies noted minimal adverse effects and high patient satisfaction, emphasizing DN as a patient‐friendly option for managing myofascial pain and TMD.

### 4.2 Comparative Therapies and Adjunctive Benefits

Other modalities such as low‐level laser therapy (LLLT), fascial manipulation, and pulsed electromagnetic fields (PEMF) also demonstrated significant improvements in pain and function, albeit with varying efficacy. Fouda's (2014) study identified PEMF as the most effective intervention for pain relief among the tested modalities, advocating for its adjunctive use to enhance myofascial trigger point inactivation.

Sekito et al. (2022) highlighted the potential of FM in providing rapid and cost‐effective improvements in pain and mouth opening. Their study indicated that FM facilitated faster recovery of function compared to conventional TMD treatments, emphasizing its utility in clinical practice. Conversely, Oliveira's comparison of LLLT and DN found both therapies effective for pain reduction, though DN was numerically superior for improving mouth opening (Oliveira et al. 2018).

### 4.3 Functional and Morphological Outcomes

Beyond pain management, DN's influence on muscle morphology and functional metrics was evident. Botticchio et al. (2021) showed significant reductions in muscle and disc thickness following DN, along with improved mouth opening, suggesting a morphological basis for its therapeutic effects. Similarly, Dib‐Zakkour et al. (2022) reported enhanced symmetry and jaw trajectory, coupled with reduced muscle activity, underscoring DN's role in optimizing functional alignment.

### 4.4 Tinnitus and Myofascial Pain

Sirikaku's (2023) study extended DN's benefits to patients with tinnitus and muscular TMD, reporting substantial reductions in tinnitus intensity and discomfort alongside pain relief. This reinforces the multidimensional therapeutic potential of DN, particularly when combined with counseling interventions.

### 4.5 Broader Implications

Ferreira et al. (2024) highlighted the complementary effects of manual therapy and DN, with both modalities significantly enhancing mandibular mobility and reducing pain over time. These findings advocate for the integration of DN with other therapeutic approaches to maximize clinical outcomes for TMD patients.

### 4.6 Adverse Effects

No major adverse effects were reported following the DN procedure. Patients who experienced side effects, such as muscle soreness, ecchymosis, headache, drowsiness, and nausea, reported that they lasted from several hours to a week (Dunning et al. 2024). Also, for alleviating the possible pain after DN, patients were instructed to apply ice or moist heat on the painful region (Oliveira et al. 2018).

### 4.7 Limitations

While this review highlights the promising outcomes associated with dry needling, some limitations must be acknowledged. First, the heterogeneity in study designs, sample sizes, and intervention protocols across the included studies limits the generalizability of the findings (Moradzadeh, Mansournia, et al. 2019). Variability in patient populations, such as differences in age ranges, comorbid conditions, and severity of TMD, may have influenced treatment outcomes (Moradzadeh, Golmohammadi, et al. 2019).

Second, many studies relied on subjective pain assessments, such as VAS and VRS, which may introduce bias. Also, CT or MRA evaluation of TMJ and its surrounding structures was not carried out in any of the included studies to assess the exact effect of DN. The lack of standardized outcome measures further complicates comparisons between studies. Additionally, follow‐up periods were often short and did not exceed more than 6 months (Sekito et al. 2022), leading to difficulty in assessing the long‐term efficacy and sustainability of dry needling's benefits (Amini et al. 2022).

Thirdly, the sample sizes in several studies were comparatively small, decreasing the statistical power and potentially overestimating treatment effects. Larger, multicenter trials with rigorous randomization and blinding are necessary to validate these findings (Hassanzadeh et al. 2012).

Finally, potential confounding factors, such as concurrent use of other therapies and variations in practitioner expertise, were not consistently controlled across studies. These factors may influence the observed efficacy of dry needling and other modalities (Moradzadeh, Mansournia, et al. 2019).

Addressing these limitations in future research will enhance the reliability and applicability of findings, paving the way for more standardized and evidence‐based approaches to TMD management.

## 5 Conclusion

The reviewed studies collectively emphasize dry needling's efficacy as a primary or adjunctive therapy for TMD, with significant benefits in pain reduction, functional recovery, and morphological improvements. Dry needling's ability to target specific trigger points and facilitate structural changes, such as reductions in muscle and disc thickness, adds to its clinical value. Its application extends beyond pain relief to improving functional alignment and addressing associated conditions like tinnitus, further highlighting its versatility.

Comparative analyses suggest DN's superiority in certain contexts; however, integrating it with other modalities, such as manual therapy, spinal manipulation, or LLLT, may yield even better outcomes. The minimal adverse effects and high patient satisfaction associated with DN reinforce its role as a safe and patient‐friendly approach to TMD management.

Future studies should focus on larger sample sizes, standardized protocols, and long‐term follow‐ups to refine these findings and establish robust, evidence‐based guidelines. The integration of advanced imaging and objective metrics may further elucidate DN's underlying mechanisms and optimize its clinical application.

## Author Contributions

**Mina Khayamzadeh:** conceptualization, methodology, database search, literature screening, data extraction, synthesis of findings, and writing – original draft preparation. **Afagh Tavassoli:** literature screening, data extraction, synthesis of findings, and writing – review and editing. **Farnoosh Razmara:** validation of literature screening and data extraction, supervision, and writing – review and editing. All authors contributed actively to the development of this systematic review, approved the final version of the manuscript, and agree to be accountable for all aspects of the work.

## Ethics Statement

Ethics approval is not applicable in this study.

## Consent

Patient consent is not applicable in this study.

## Conflicts of Interest

The authors declare no conflicts of interest.

## Data Availability

Data sharing is not applicable to this article as no new data were created or analyzed in this study.
